# Quantitative analysis of transmission parameters for bluetongue virus serotype 8 in Western Europe in 2006

**DOI:** 10.1186/1297-9716-42-53

**Published:** 2011-03-24

**Authors:** Aline A de Koeijer, Gert Jan Boender, Gonnie Nodelijk, Christoph Staubach, Estelle Meroc, Armin RW Elbers

**Affiliations:** 1 Department of Epidemiology, Crisis management and Diagnostics, Central Veterinary Institute (CVI), part of Wageningen UR, P.O. Box 65, NL-8200 AB Lelystad, The Netherlands; 2 Friedrich-Loeffler Institut, Institute of Epidemiology, Wusterhausen, Germany; 3 Veterinary and Agrochemical Research Centre (VAR-CODA-CERVA), Brussels, Belgium

## Abstract

The recent bluetongue virus serotype 8 (BTV-8) epidemic in Western Europe struck hard. Controlling the infection was difficult and a good and safe vaccine was not available until the spring of 2008. Little was known regarding BTV transmission in Western Europe or the efficacy of control measures. Quantitative details on transmission are essential to assess the potential and efficacy of such measures.

To quantify virus transmission between herds, a temporal and a spatio-temporal analysis were applied to data on reported infected herds in 2006. We calculated the basic reproduction number between herds (*R_h_*: expected number of new infections, generated by one initial infected herd in a susceptible environment). It was found to be of the same order of magnitude as that of an infection with Foot and Mouth Disease (FMD) in The Netherlands, e.g. around 4. We concluded that an average day temperature of at least 15°C is required for BTV-8 transmission between herds in Western Europe. A few degrees increase in temperature is found to lead to a major increase in BTV-8 transmission.

We also found that the applied disease control (spatial zones based on 20 km radius restricting animal transport to outside regions) led to a spatial transmission pattern of BTV-8, with 85% of transmission restricted to a 20 km range. This 20 km equals the scale of the protection zones. We concluded that free animal movement led to substantial faster spread of the BTV-8 epidemic over space as compared to a situation with animal movement restrictions.

## Introduction

On August 15^th^, 2006, Western Europe was alerted to the presence of Bluetongue virus serotype 8 (BTV-8) in The Netherlands [1]. Later it turned out that the infection had been present in the area around the borders of Belgium, The Netherlands and Germany for several weeks, and the infection was found to have already spread throughout a large area with a 200 km radius around the focus point [2]. The route of introduction remains unknown, although various suggestions have been studied [3].

Various control measures like animal transport restrictions, use of insecticides and moving the animals in house, were introduced [4], but these options all appeared to have limited effect. It was suggested that the winter season of 2006/2007 would halt the BTV epidemic, assuming that the chain of transmission would be broken by a stop in the life cycle of the vector because of low temperatures. However, during 2007 it became evident that BTV-8 had survived the winter in Western Europe and a re-emerging epidemic quickly developed within the originally affected countries, affecting approximately 40 000 locations with ruminants [5-7]. In addition, BTV-8 was introduced into the United Kingdom, Denmark, Switzerland and the Czech Republic. As a response, the EU Commission recommended vaccination as the most efficient veterinary measure that may be used to fight BTV-8.

The veterinary pharmaceutical industry made a great effort by developing an inactivated vaccine against BTV-8 in slightly less than two years [8]. This vaccine has been applied in the affected region since May 2008. The vaccine is more effective than all the initial attempts of controlling or isolating the problem, measured in terms of new clinical outbreaks in the affected countries in 2008 [5,6,9].

At the time, little knowledge was available about the dynamics of this infection in the host and the vectors, and also about the dynamics of the vectors themselves. The limited effect of many early measures to control the epidemic are a result of this knowledge gap. Publications on the transmission dynamics of BTV in this region became available soon [10,11]. However, due to lack of data, these studies were still based on general information on BTV and *Culicoides. *They incorporate specific BTV transmission data from other regions and other BTV-strains and only incorporate specific information on the actual West-European situation on well known variables such as temperature and host densities. During this epidemic, studies have been initiated to learn more about the abundance and dynamics of the vectors and their potential for transmitting BTV in this area [12-16].

To learn more about the quantitative aspects of the dynamics of the infection, the epidemic data from the recent epidemic are very helpful. Thanks to good collaborative efforts amongst the countries involved, we were able to jointly analyse the data from the whole affected area in 2006. The availability of background information on herds without BTV was also essential.

Spatial and temporal methods were used to quantify the BTV-8 transmission between herds in Western Europe in that period and to evaluate the effect of the changing seasons (temperature) on this transmission.

## Materials and methods

### Data and data handling

In the framework of the European FP6 Network of Excellence of Diagnostics and Control of Epizootic Diseases Epizone (see [17]), a project has been granted for international collaboration in Bluetongue (BTV-8) Epidemiological research (Internal Call Work Package 6.6). In this project, National Reference Laboratories from Germany (FLI), The Netherlands (CVI) and Belgium (VAR-CODA-CERVA), together with the Centre de Coopération Internationale en Recherche Agronomique pour le Développement (CIRAD), Montpellier, France worked on an epidemiological analysis of the BTV-8 epidemic in ruminant herds in 2006.

The epidemiological data we used were the following: the geographical coordinates for each outbreak holding (in total the dataset includes 1977 reported farms, half of which are cattle farms, almost half are sheep farms and a few mixed farms); the date of clinical suspicion reported to veterinary authorities and the date of first BTV-associated clinical signs observed within the outbreak holding. Furthermore we used information on the timing of control measures implemented by each country and information on the density of all holdings housing cattle, sheep and goats per community with a variable level of detail. FLI provided a secure database platform and server making these and some further background data on the 2006 BTV-8 epidemic available to all group members.

Temperature data for the epidemic period was obtained from the Royal Dutch Meteorological Institute (KNMI).

### Transmission modelling

The above mentioned data were used to quantify the transmission between herds (1) in terms of a reproduction number (only temporal aspects of the affected herds were used) and (2) in terms of a spatial transmission kernel (both spatial and temporal aspects required). The first method was used to evaluate the impact of seasonality and temperature on transmission, while the second method aimed at determining the spatial scale of BTV-8 transmissions.

#### Basic reproduction number

We analysed the effect of seasonality in the transmission of BTV-8 using a method to quantify *R_h_*, the basic reproduction number between herds. This method was used previously to estimate *R_h _*based on epidemic data per herd for the epidemic of Foot and Mouth disease (FMD) in The Netherlands in 2001 [18]. The basic reproduction number is defined as the expected number of new infections, which are generated by one initial infective subject (animal or herd). Thus, it is by definition a measure of the transmission per infection generation. The practical use of this number lies mainly in the threshold behaviour when the reproduction number equals one. If the number is lower than one, an epidemic cannot occur, because the number of new infections decreases per generation. If the number is higher than one, the population is at risk of an epidemic, but it will not necessarily happen. Essential parameters in this method are the time (date) at which the infection is introduced in the herd, the time (date) the herd becomes infectious to the surrounding herds and the end of the infectious period.

For the BTV-8 epidemic it was rather difficult to determine the time of introduction of the infection and the infectious period. Therefore, we started using a few basic assumptions to solve this, followed by a more detailed sensitivity analysis to evaluate the impact of an assumption with a strong influence. We assume that a herd becomes infectious at the time when the first clinical symptoms were observed (i.e. when several infectious animals are around). We considered that it will take about two weeks before the infection has spread substantially in a herd. We assumed a latent period of 14 days, i.e. the introduction of the infection in the herd took place 14 days earlier. Since the infection can spread and persist in livestock and vectors in and on the farm for several months, we also assume that all infected farms remain infectious during the whole vector-active season. Similar assumptions have been applied previously by Szmaragd et al. [19].

We analysed the full data set of the 2006 epidemic under these assumptions. To assess if there was a regional effect on transmission we also analysed the data from Belgium, Germany and The Netherlands separately. Furthermore, we tested the impact of the assumption concerning the infectious period. This assumption was rather crude, so we also calculated the reproduction number assuming shorter infectious periods.

#### Kernel estimation

The spatio-temporal analysis applied a method as published by Boender et al. [20,21]. In this methodology, the transmission kernel is described as a transmission rate *λ*(*r*) over distance (*r*). To be as general as possible, while limiting the number of possibilities we used a functional shape of the class(1)

in which, *r *is the inter-farm distance, *λ_0 _*is the initial rate of transmission and *r_0 _*a scaling distance. Via the power α the total range of global (α < 2), intermediate (2 < α < 3) and local kernels (α > 3) could be matched. Boender et al. [21] used the moments of the spatial kernels to characterise them. For global kernels the transmission is not limited to certain regions, because average transmission distance, i.e. the first moment, is infinite. For intermediate kernels the transmission could be regional depending on the actual location of farms in a country, because the average transmission distance in one dimension exists, while the average transmission distance in two dimensions, i.e. the second moment, is infinite. For local kernels the transmission is regional, because the average transmission distance in two dimensions exists. The kernel shown in equation (1) gives the best fit describing the FMD outbreak in The Netherlands in 2001 and the Avian Influenza epidemic in 2003 [20,22]. We assumed in this formalism that the transmission is isotropic (independent of direction), homogeneous (independent of location of the farm) and constant (time independent during the infectious period). The transmission probability for an infectious period *T *and an inter-herd distance *r *equals:.

Using a Maximum Likelihood (ML)-estimation [20] the parameter estimation for the 2006 BTV-8 epidemic in Europe could be obtained and the detailed results are presented in Table 1. Because control measures were not uniform in the different countries we split the 2006 dataset and we performed the ML-estimation for the different countries separately. It was difficult to estimate the parameters *λ_0 _*and *r_0 _*for the Belgium transmission rate with a meaningful confidence interval (CI), because these parameters appeared to be interdependent. Therefore we reduced the transmission rate for Belgium to(2)

**Table 1:** Maximum Likelihood (ML)-estimates for the transmission rate parameters during the bluetongue virus serotype 8 epidemic in 2006 for the different areas of interest (confidence intervals within brackets)

Regions	Function	λ_0_(10^-6 ^day^-1^)	r_0_(km)	α
Europe		7.4 (5.6-10)	8.8 ( 7.0-10.9)	2.5 (2.3- 2.6)
Germany		9.2 (6.6-13.4)	18.0 (13.5-23.0)	3.2 (2.9- 3.7)
Netherlands		24. (16-52)	3.9 ( 2.1- 6.1)	2.0 (1.9- 2.2)
Belgium before 24/08/2006		62. (35-161)	17.5 (8.3-26.8)	3.7 ( 2.6-5.9)
Belgium		λ_0_r_0_^α^(m^α^day^-1^) 0.028 (0.012-0.06)		1.1 (1.0-1.2)
Belgium after 24/08/2006		0.0045 (0.0009-0.017)		0.96 (0.8-1.2)

with only two degrees of freedom {*λ*_0_*r*_0_^*α*^,α}.

## Results

### Reproduction number and temperature influence

**Figure 1:**
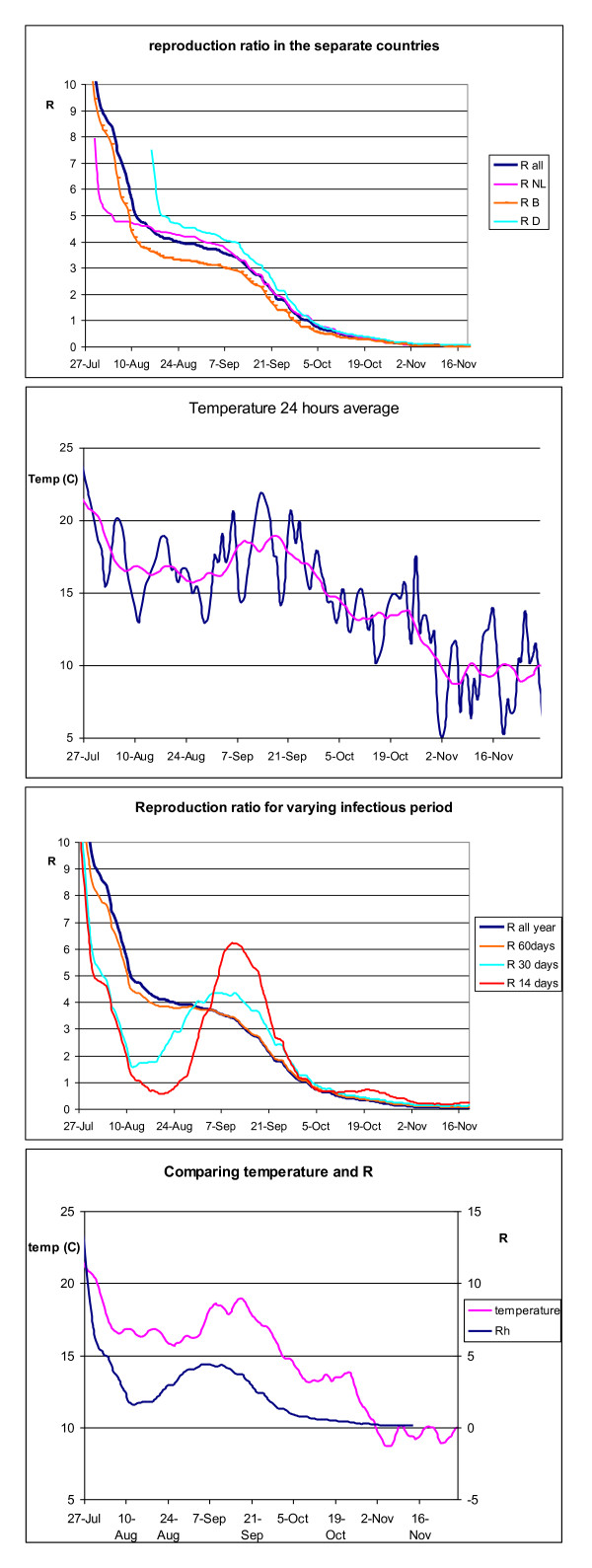
**Figure 1a. The development of the basic reproduction number (measure of transmission between herds) *R_h _*over time during the bluetongue virus serotype 8 epidemic in 2006 for Germany (D), Belgium (B) and The Netherlands (NL and for the whole area (all)**. The date given is the start of the infectious period. Figure 1b. Average temperature per twenty-four hours (blue) during summer and fall 2006 (in°C) and the 14 days rolling average (purple) for a smoothened temperature curve. Figure 1c. Basic reproduction number of BTV-8 (between herds) in a sensitivity analysis for various infectious periods. The date given is the start of the infectious period. Figure 1d. Joint view of the temperature (14 days rolling average) and the estimated reproduction ratio assuming a 30 days infectious period.

We estimated the reproduction number for BTV-8 in the second half of the summer to be around 4, declining below 1 in the fall. The detailed results of the temporal analysis, calculating the effective *R_h _*in the field, are given in Figure 1a. These results show the effect of the temporal changes in the BTV epidemic. For each infectious herd the graph shows the estimated number of new herds that were infected by this source herd. Thus we obtained an estimate for *R_h _*during the summer and fall season. We found that the epidemic threshold, *R_h _*= 1, is passed at the beginning of October. Since then, the 2006 epidemic declined. This analysis was done separately on the full dataset and on the data from the three most affected countries (Belgium, Germany and The Netherlands). The results for Belgium indicate that *R_h _*declines below 1 two days earlier than in the other two countries, while the estimated reproduction number declines below 1 at the same day as in the complete epidemic. Overall we found very little difference between the countries in the level of transmission between herds (Figure 1a).

In all countries, focussing on the first few weeks of the epidemic, very high *R_h _*estimates were obtained. Although the very warm period in July 2006 will surely have led to a high reproduction number between animals, this will be an overestimation because the infectious herds in that period were few in number, there was a large impact of underreporting, and these farms were assumed to have a very long infectious period.

A sensitivity analysis was performed, to study the effect of assuming different lengths of the infectious period (see Figure 1c). We hypothesise that in most herds the majority of the infection pressure will be emitted in a few weeks up to at most two months, depending on temperature and *Culicoides *density. Therefore, we also studied several shorter infectious periods between two weeks and two months. The analysis shows that the assumed temperature effect (compare with Figure 1b) becomes more pronounced with shortening of the infectious period.

There is a strong parallel between average temperature and *R_h _*(Figure 1a-d), compare especially Figure 1b and 1c. July 2006 was extremely warm, with daily average temperatures over 20°C (normal temperatures would be around 17 or 18°C). The temperature declined in the first week of August, and remained rather constant for a long time. The average temperature was about 16°C in August and about two degrees higher in September. During October the average temperature started to decline again to normal values for that time of the year of around or below 10°C in November. This pattern is very similar to that of *R_h_*: very high in July (*R *> 5) and remaining almost constant during August and September (*R_h _*≈ 4) and declining after that. In the graphs that depict *R_h _*under a shorter assumed infectious period, the effect of lower average temperatures in August and higher average temperatures in September is also reflected (Figure 1c).

When the infectious period ranges between one and two months, we found that the warm period in July still has high *R_h _*estimates, i.e. larger than 5. In the less warm period of August and September, *R_h _*is rather constant at about 4 new herds for each herd that became infectious in that period.

Although a very short infectious period of two weeks may not be realistic for most of the season, the analysis under that assumption helps us in visualising the strong effect of the temperature on the level of transmission (see Figure 1c). The impact of a very small temperature blip at the end of October can actually be observed in the estimated transmission in those weeks. Thus we can more precisely estimate the threshold temperature at which the epidemic switches from increase to decline. We find that this threshold temperature lies between 14 and 16°C. Some variance in the temperature effect during the autumn can be expected from declining numbers of *Culicoides *in the autumn period [23].

We found that the moment at which the threshold is passed, is not sensitive to the assumed length of the infectious period at all. In all analyses, *R_h _*declines below 1 around September 20^th ^(the date of introduction of the infection in a herd). This differs two days at most over the various analyses performed. This last aspect suggests that indeed the infection pressure is mainly spread out in a few weeks after the estimated start of the infectious period. Otherwise, we should have observed a delay between changes in temperature and changes in the *R_h_*.

### Spatial transmission kernel

**Figure 2:**
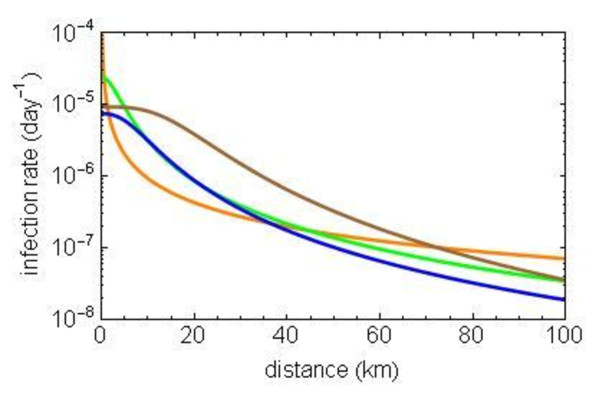
**Spatial transmission kernel of the BTV-8 epidemic in 2006 in Belgium (orange), The Netherlands (green), Germany (brown) and the complete Western European area (blue)**.

The spatio-temporal analysis, which calculated the spatial transmission kernel, used the same basic assumption of a latent period of 14 days and an infectious period lasting until the end of the year. The results are shown in Figure 2. From earlier studies we know that the transmission of FMD and AI between farms in The Netherlands takes place over a spatial scale (i.e. defined as the first moment by Boender et al. [21]) of respectively 2 and 4 kilometers [21,22]. We found that BTV-8 in 2006 spreads over a much larger spatial scale of about 15 km. Eighty-five percent of all transmission takes place within a 20 km range, which equals the size of the restriction zones.

In Figure 2 we also show the results of the same analysis on data for the three most affected countries separately. The Belgian kernel did not fit well in a three parameter kernel shape. A reduced two parameter kernel turned out to be more suitable for this situation, and it was used according to equation (2). The heavy tail of the kernel in the Belgian epidemic must be noted here. Clearly, long distance transmission was far more important in this country than it was in the other two countries. A possible explanation was found in the restriction zoning. On August 24^th^, 2006, Belgium declared the whole country to be a BTV-8 infected area. Since that date, all livestock transport within Belgium was free from restrictions again. All livestock (infected or not) could be moved freely throughout the whole country. In contrast, in The Netherlands and Germany, with every new animal holding declared infected, gradually growing restriction zones were redefined regularly. As a result, considerable parts of these countries were not incorporated in the restriction zones and animals could not be moved from the infected area to free areas unless tested by a laboratory diagnostic test.

**Figure 3:**
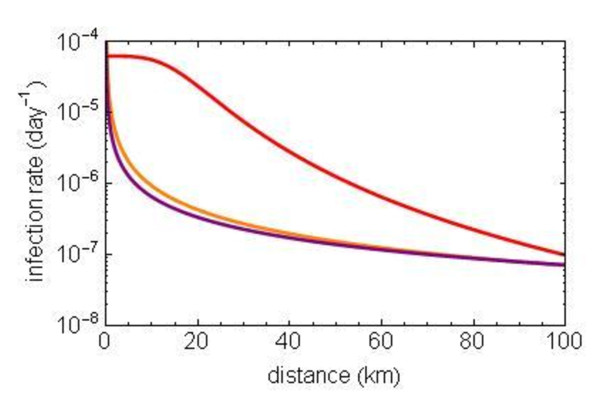
**Spatial transmission kernel of the bluetongue virus serotype 8 epidemic in 2006 in Belgium (orange), and separately for the first weeks until August 24^th ^(red) and in a later stage without transport restrictions (purple)**.

To assess the effect of this difference in the control measures in Belgium, we separately analysed the period before August 24^th^, and the period thereafter. The first obvious result was that the earlier period shows a higher level of transmission than the second period (Figure 3). This fits with our results about the strong effect of temperature on the transmission of BTV-8, which was confirmed in the *R_h _*results in this paper. The second result from this analysis was found in the relative distribution of long and short distance transmissions in the two periods. The first period until August 24^th ^shows much more short and medium distance transmissions, whereas the kernel for the second period (after August 24^th^) in Belgium shows an almost horizontal tail of the transmission kernel over space. The latter means that the transmission can hardly be distinguished from a random distribution of the infection over space (little decline of the infection rate over a longer distance). Thus, in the earlier period, there is a clear spatial transmission kernel for Belgium that has the same shape as the kernels in the other two countries, whereas there is little spatial effect left in the transmission kernel for the period after August 24^th ^in Belgium.

The main gain of the collaboration between the affected countries, leading to a joint analysis of the data gathered, was found in the results from the spatial analysis. When all data of the affected area were analysed together, we found that the estimated long distance transmission (in the range of 50 to 100 km) is lower than that found in an analysis of the separate countries. In analyzing the data from one country separately, some transmissions need to be assumed to have originated much further away, than the near-by infected farms over the border, leading to an overestimation of long distance transmission.

## Discussion

From the complete data set of the BTV-8 infected area in 2006, we estimated the reproduction number between herds, *R_h_*, during the BTV-8 epidemic in 2006 to be around 4 in the second half of the summer. This part of that summer was slightly less warm than usual followed by a warm September. The reproduction ratio declined below 1 in the fall. This pattern was also consistent for the three countries that were analysed separately. We are the first to quantify these parameters for such an infection, so these results cannot be compared to earlier publications. The differences found between the countries are surprisingly small, which means that the essential features driving the epidemic were comparable throughout the affected region. The reproduction number between herds was of the same order as that found for major dreaded livestock infections, like FMD [18,24].

This epidemic was able to hit most ruminant herds in a large area, which is unprecedented for notifiable diseases in Western Europe in recent decades. This huge impact was a result of a lack of available effective control measures in 2006. In FMD and CSF epidemics, isolation of infected herds immediately after detection in the form of a stand-still or transport ban, leads to a major reduction of transmission, bringing *R_h _*close to or below 1 depending on local conditions. [22,25]. However, for a vector-borne disease, national veterinary authorities in the affected area assumed a ban on animal transport to be less effective and therefore these restrictions were not applied in the rigorous way that is common for other notifiable diseases like FMD or CSF.

From the sensitivity analysis, we learned that the *R_h _*estimates are not very sensitive to assumptions on the duration of the infectious period of a herd. It remains in the same range in all cases. This analysis also visualises the effect of temperature on transmission. Temperature is the only really obvious connection explaining the decline of the epidemic in the autumn, which fits with predictions from earlier theoretical modelling studies [10]. The decline of *R_h _*during the season cannot be explained by increasing the efficacy of the control measures, because none were newly introduced during the period when the transmission declined in the autumn. The temperature is thought to affect many aspects of the vector-host transmission system, like the intrinsic incubation period in the vector, the biting rate and lifespan of the vector, and with some more delay also the density of the vector population. All these aspects lead to reduced transmission at lower temperatures.

By studying the results for extremely short infectious periods for a herd, we were able to visualise the impact of temperature on such a system. The number calculated in this way may led to a less precise quantification of reproduction number between herds, but it surely is a good method for visualising the impact of temperature on transmission. We found that a few degrees temperature decline in the range between 15 and 20°C can lead to a reduction of transmission up to a factor 10. This is a substantial difference in transmission as a result of a rather common temperature range difference in Western Europe. Thus, the transmission appears to be extremely sensitive to this aspect, as was previously expected but never proven [10,11]. It also supports findings that the infection has more difficulty invading Scandinavia, where summer temperatures are slightly lower.

We also determined a spatial transmission kernel of the infection between herds and found that although for The Netherlands the shape of the spatial transmission kernel for BTV-8 is quite similar to those found in earlier studies on AI and FMD [21,22], the spatial scale at which transmission for BTV-8 took place, was much higher. Where transmission of FMD is mostly restricted to a few kilometers, BTV-8 easily spreads over about 15 km ranges. This difference probably follows from two typical features of the BTV-8 epidemic. First of all, unlike FMD and AI, there were no attempts to control this infection with a strict ban on animal transport, which is a common regulation for FMD and AI epidemics. Secondly, the vector can easily move around several kilometers, and more if helped by wind [26]. Thus, longer distance transmission of this infection is to be expected. The effect of transmission via the wind, leading to an asymmetrical spatial spread is not included in the model. During the summer and fall of 2006, the wind direction was quite variable, leading to a very diffuse pattern throughout the infectious period. A specific analysis of the effect of wind in the 2006 BTV-8 epidemic can be found in Hendrickx et al. [26].

In the analysis over separate countries, by not allowing transmission over the border, we forced long distance transmission into the analysis. These extra long distance transmissions are not needed in a joint analysis of the complete infected area. Thus, the latter gives a more reliable estimate of the long distance transmission averaged over the duration of the outbreak and all the countries involved.

Still, the shape of the spatial kernel we found in analysing the overall area is very different from the Gaussian kernel for BTV transmission estimated from the same epidemic by Szmaragd et al. [19,27]. This is because their analysis, which uses the same case dataset, only incorporates reported farms, and ignores the background density of farms present in the area. To regard their study result as a transmission kernel, a uniform density of background farms have to be assumed. Since the background of uninfected and undetected farms are not at all uniformly distributed, this assumption leads to an overestimation of the number of farms in low density regions and consequentially to an underestimation of the number of farms in high density regions. In a kernel estimation for case data, this leads to an underestimation of the probability of long distance transmission and thus to a too much localised kernel estimate.

In Belgium, we found a spatial transmission pattern in 2006 that diverges substantially from that in The Netherlands and Germany. We observed a very heavy tail of long distance transmission in Belgium, suggesting a restricted influence of the spatial component in transmission in Belgium. The kernels for The Netherlands, Germany and the kernel for the whole area show a much more localised spatial transmission.

A separate analysis for the early (with animal movement restrictions) and late period (no animal movement restrictions) of the epidemic in Belgium showed a major difference between the spatial transmission kernels before and after August 24^th^, 2006. This suggests that unrestricted animal transport has a major impact on the spatial scale of transmission of this infection. It leads to a transmission pattern that is similar to random transmission over space in the area where such transports are allowed freely.

All other countries maintained restrictions on animal transports from the more heavily infected areas, to protect the zone that was not yet affected (heavily), thus reducing long distance transmissions and maintaining a pattern of gradual spread over space. The scale of the spatial transmission kernel fits with the size of the restriction areas (i.e. average transmission distance of 15 km). According to Szmaragd et al. [27] for the Gaussian kernel, movement restrictions had only a small effect on spread, whereas for fat tailed kernels, such restrictions lead to greatly reduced spread. We observed a large effect of the movement restrictions on the spatial spread, which was consistent with a fat tailed kernel and inconsistent with the Gaussian kernel selected by Szmaragd et al. [19].

Therefore, our results suggest that further restriction of animal movements could have helped in reducing long distance transmission. Such an intervention did not affect the short distance transmission and did not change the intensity (prevalence) of the epidemic in affected areas, but slowed down the spatial progress (speed of the wave front) of the epidemic. This can be of major importance in protecting neighbouring countries for introduction of the infection.

Finally we add a few remarks on the results to make our conclusions more informative. Firstly, in this study we were able to analyse transmission parameters only on the level of transmission between herds. This follows from the type of data that were collected, i.e. estimated date of infection and location of each infected farm. Within herd information was very limited and will be analysed separately. Although effective transmission within a herd will probably continue later in the season than the transmission between herds, the main spread of the epidemic was already restricted early in the autumn, because transmission between herds had declined below the threshold. Transmission between herds is the essential feature of large scale epidemics in livestock.

Secondly, underreporting and late reporting was probably substantial in this epidemic. If the underreporting was constant throughout the assessed period, this did not influence the amplitude of the spatial kernel, while the ratio between infected and uninfected was consistently underestimated. It will not have influenced our *R_h _*results or the shape of the kernel much. This is because the transmission (for both methods applied here) was quantified from the ratio between infectious and newly infected farms which are registered. Thus it missed a similar fraction of farms on both ends, the infectious and the infected end.

Thirdly, the Culicoides densities were unknown at the time of the study. Since then it has become clear that suitable vector species for BTV-8 are prevalent throughout the area [12-16]. Details on the vector abundance and vector competence for the various *Culicoides *species, was not available in sufficient detail to analyse its influence.

In conclusion we found that

1. animal transport restrictions can slow down the spatial spread of BTV-8 substantially.

2. spatial transmission during the BTV-8 epidemic took place mostly within a 20 km range.

3. at temperatures below 15°C, the transmission of BTV-8 between farms was limited to such a low level that the epidemic was fading out.

4. the reproduction number of BTV-8 between herds is about 4 in a normal summer in Western Europe.

## Competing interests

The authors declare that they have no competing interests.

## Authors' contributions

AdK was responsible for the data analysis, modelling, visualisation and main conclusions regarding the temporal analysis with links to the temperature effect, and wrote the majority of the paper. GJB was responsible for the data analysis, modelling, visualisation and main conclusions of the spatio-temporal analysis, and wrote most of the materials and methods and results regarding these aspects. GN and AE helped the analysis forward in brainstorm sessions regarding results and conclusions, and contributed to the preparation and finalisation of the paper. AE was also responsible for the Dutch data and was coordinator of the international collaboration on BTV-8 epidemiology in Epizone. CS was responsible for the German data, for the overall data structuring, inspection and management. He also offered information on the German epidemiological situation regarding BTV-8. EM was responsible for the Belgian data and the background information regarding the epidemiological situation of BTV-8 in Belgium. All authors read and approved the final document.
